# Electrochemical Detection of Alzheimer’s Disease Biomarker, β-Secretase Enzyme (BACE1), With One-Step Synthesized Reduced Graphene Oxide

**DOI:** 10.3389/fbioe.2022.873811

**Published:** 2022-03-24

**Authors:** Jhilik Dey, Akanksha Roberts, Subhasis Mahari, Sonu Gandhi, Prem Prakash Tripathi

**Affiliations:** ^1^ Cell Biology and Physiology Division, CSIR-Indian Institute of Chemical Biology, Kolkata, India; ^2^ Cell Biology and Physiology Division, IICB-Translational Research Unit of Excellence, Kolkata, India; ^3^ DBT-National Institute of Animal Biotechnology (NIAB), Hyderabad, India

**Keywords:** Alzheimer’s disease, BACE1, graphene oxide, immunosensor, diagnostic

## Abstract

β-Secretase1 (BACE1) catalyzes the rate-limiting step in the generation of amyloid-β peptides, that is, the principal component involved in the pathology of Alzheimer’s disease (AD). Recent research studies show correlation between blood and cerebrospinal fluid (CSF) levels of BACE1 with the pathophysiology of AD. In this study, we report one-step synthesized reduced graphene oxide (rGO), activated *via* carbodiimide chemistry, conjugated with BACE1 antibody (Ab), and immobilized on fluorine-doped tin oxide (FTO) electrodes for rapid detection of BACE1 antigen (Ag) for AD diagnosis. The synthesis and fabrication steps were characterized using different types of spectroscopic, X-ray analytic, microscopic, and voltametric techniques. Various parameters including nanomaterial/Ab concentration, response time, pH, temperature, and rate of scan were standardized for maximum current output using the modified electrode. Final validation was performed *via* detection of BACE1 Ag ranging from 1 fM to 1 µM, with a detection limit of 0.64 fM in buffer samples and 1 fM in spiked serum samples, as well as negligible cross-reactivity with neurofilament Ag in buffer, spiked serum, and spiked artificial CSF. The proposed immunosensor gave a quick result in 30 s, and good repeatability and storage stability for a month, making it a promising candidate for sensitive, specific, and early diagnosis of AD. Thus, the fabricated electrochemical biosensor for BACE-1 detection improves detection performance compared to existing sensors as well as reduces detection time and cost, signifying its potential in early diagnosis of AD in clinical samples.

## Introduction

Alzheimer’s disease (AD) is a chronic neurodegenerative ailment that affects adults in the later stage of life. It is caused by memory and cognitive impairment, combined with gradual neuronal death (Hodson, 2018). AD pathogenesis is characterized by neuropathological conditions including formation and deposition of extracellular amyloid-beta (Aβ) aggregates [a 39-42 amino acid long peptide produced *in vivo via* specific, proteolytic division of amyloid precursor protein (APP)] and formation of neurofibrillary tangles due to accumulation of intracellular hyperphosphorylated tau proteins (microtubule-associated protein expressed in neurons for functioning of cytoskeletal network in terms of microtubule assembly) (Mohd Sairazi and Sirajudeen, 2020). β-Site amyloid precursor protein-cleaving enzyme 1 (BACE1), also called β-secretase1, is a transmembrane aspartyl protease type I (hence termed as enzyme 1) that is expressed in the brain, specifically in neurons and glia. BACE1 cleaves APP and acts as a rate-limiting step for Aβ production. BACE1 protein concentrations (normal concentration = 16 pg/ml) (Mulder et al., 2010) and activity were quantified in cerebrospinal fluid (CSF) to examine its association with amyloid-β pathway, neurodegeneration, synaptic dysfunction, and pathophysiological changes (Hampel et al., 2021). Various studies have proposed BACE1 as a potential AD- and dementia-specific biomarker. Recent studies have found that the BACE1 level is linked with Aβ and tau markers. BACE1 activity was significantly higher in individuals with AD characteristics than in healthy controls (normal concentration = 16 pg/ml and AD concentration = 20 pg/ml) (Mulder et al., 2010). Along similar lines, reduced BACE1 in CSF was observed in individuals having mild cognitive impairment (MCI) without AD pathophysiology (16.10 pM) than in individuals having MCI with AD pathology (19.28 pM) (Alexopoulos et al., 2018). Expression and activity of BACE1 was also measured in CSF of a deceased AD patient’s brain (Thambisetty and Lovestone, 2010; O’Bryant et al., 2016).

Recent studies have also used blood BACE1 as a predictive marker for AD and dementia. Indeed, BACE1 was higher in the plasma of individuals with AD than in healthy age match controls (normal biomarker profile = 3.5 pM and AD biomarker profile = 25 pM) (Wu et al., 2012). Additionally, during a longitudinal study, plasma BACE1 activity was higher in individuals with MCI that advanced to AD than those that did not advance (AD patients ≥2.6 mFU/min/μg and healthy control 2 to 6 mFU/min/μg) (Shen et al., 2018). These results indicate that BACE1-mediated Aβ accumulation starts many years before the onset of AD, thus advocating the promising role of BACE1 as a reliable biomarker for early detection of AD, especially in serum (Cervellati et al., 2020).

Most of the methods that detect BACE1, namely, enzyme-linked immunoassays and polymerase chain reaction, are time-consuming, require skilled personnel, and are not point-of-care diagnostics with low detection limits. Thus, developing a biosensing system has significant importance for personalized health care of AD individuals. Biosensors make use of various biomarkers (antibodies, enzymes, aptamers, ligands) to gather information regarding some biological, chemical, or physical change and then transform the information into a readable signal. Electrochemical sensors have been considered to be a promising tool due to their fast response time and ability for real-time and on-site detection by generation of an electrochemical signal, and a few have been developed for detection of different AD biomarkers such as acetylcholine enzyme (Chauhan et al., 2019; Chauhan et al., 2020) and certain proteins (Esteves-Villanueva et al., 2014; Bungon et al., 2021).

A two-dimensional hexagonal structure of graphene contains sp^2^-hybridized carbon bonds, which are responsible for the conductive properties of graphene due to a delocalized network of electrons, and furnishes the feeble bonding among layers of graphene or in-between substrate and graphene layers (Geim and Novoselov, 2007; Hass et al., 2008). These structurally distinctive attributes give graphene and its derivatives various tunable properties such as excellent conductivity (can range from 10^4^ to 10^5^ S/m) (Stankovich et al., 2006), broad surface area, and increased mechanical strength, making it an ideal signal-enhancing nanomaterial for fabrication of sensitive electrochemical biosensors (Huang et al., 2011), and hence has been selected over other nanomaterials such as gold and silver nanoparticles. Graphene oxide (GO) and reduced graphene oxide (rGO) have an advantage over pristine graphene, in terms of the tunability in optical and electrical properties, due to additional functional groups. In the oxidized state, GO has less stability due to reactive oxygen groups present, which makes it non-ideal for fabrication of conductance-based biosensors due to electrical insulation and instability (Papageorgiou et al., 2015). However, the reduction of GO to rGO will increase double-bonded carbon atoms, which will then restore the conductivity and remove reactive oxygen sites, making it more stable and leave only carboxyl groups, which can be activated for bioconjugation. Hence, rGO with its chemically active defective sites is preferred as a signal enhancer in the fabrication of electrochemical sensors (Robinson et al., 2008). Currently developed immunosensors are sensitive, can be easily stored, are user-friendly, are rapid, and can be customized to detect a specific analyte which includes detection of cancers (Roberts et al., 2019), pesticides (Shrikrishna et al., 2021), narcotic drugs (Singh et al., 2017), bacteria (Mahari and Gandhi, 2022), and viruses (Roberts and Gandhi, 2020; Roberts et al., 2020; Roberts et al., 2021b; Roberts et al., 2021a). Graphene-based sensors have the advantage of being cheaper than other nanomaterials (e.g., gold), while remaining highly sensitive (Shahdeo et al., 2020; Narlawar and Gandhi, 2021).

In this research work, we have reduced GO to rGO, followed by carbodiimide chemistry activation and coated activated rGO on fluorine-doped tin oxide (FTO) slides, which shows higher chemical stability, electrical conductivity, and reduced physical abrasions than indium tin oxide (ITO) electrodes. BACE1 antibody (Ab) was immobilized on the activated rGO to modify the FTO sensor for BACE1 antigen (Ag) detection. All steps from coating to fabrication were thoroughly characterized using different types of spectroscopic, X-ray analytic, microscopic, and voltametric techniques. To detect BACE1 Ag in buffer samples and spiked serum ranging from 1 fM to 1 µM, the proposed electrode showed a detection limit of 0.64 fM (buffer) and 1 fM (serum). In addition to the low limit of detection (LOD), the sensor detected minimal cross-reactivity against neurofilament (NFL) Ag in buffer as well as spiked serum and artificial cerebrospinal fluid (CSF) samples, a quick response in 30 s, storage stability for 1 month, and good repeatability of electrode up to 4 times. Hence, this biosensor can be applied for miniaturization of a quick, sensitive, and specific detection kit for BACE1 protein for AD diagnostics in clinical serum samples.

## Materials and Methods

### Reagents

Sodium chloride (NaCl) was obtained from CDH (New Delhi, India). Sodium dihydrogen phosphate-1-hydrate (Na_2_HPO_4_.H_2_O) and monosodium phosphite (NaH_2_PO_3_) were acquired from Merck (India). Sodium bicarbonate (NaHCO_3_), sodium carbonate anhydrous (Na_2_CO_3_), potassium dihydrogen orthophosphate (KH_2_PO_4_), magnesium chloride (MgCl_2_), sodium citrate tribasic dehydrate (C_6_H_5_Na_3_O_7_.2H_2_O), potassium ferri- and ferro-cyanide (K_3_Fe(CN)_6_ and K_4_Fe(CN)_6_·3H_2_O) were purchased from SRL (India). 1-ethyl-3-(3-dimethylaminopropyl) carbodiimide (EDC), N-hydroxysuccinimide (NHS), and graphene oxide (GO) were procured from Sigma (India). β-Site amyloid precursor protein-cleaving enzyme 1 (BACE1) antigen (cat: 931-AS-050) and antibody (cat: MAB9311) were acquired from R&D Systems (Minnesota, United States). Human neurofilament (NFL) antigen (cat: ab224840) was purchased from Abcam (Massachusetts, United States). Chemicals, reagents, and solvents used in this research were of analytically graded high quality.

### Apparatus

Raman spectroscopy and Fourier-transform infrared (FT-IR) spectroscopy were performed on Thermo Scientific-Nicolet 6700 Raman Spectroscope and iS50 FT-IR (Bangalore, India), respectively. Surface morphology was visualized *via* scanning electron microscopy (SEM), and elemental composition was analyzed *via* energy dispersive X-ray (EDX) on ZEISS EVO SEM coupled with SmartSEM software (Germany). Cyclic and differential pulse voltametric experiments were carried out on PalmSens4 potentiostat from PalmSens (The Netherlands).

### Synthesis and Bioconjugation Characterization of rGO and BACE1 Ab

rGO was synthesized by removing oxygen from GO (Habte et al., 2019; Roberts et al., 2022). Here, the reducing agent used was ascorbic acid (AA). First, 4 g AA was added to 100 μg/ml GO under constant stirring at 60°C and later centrifuged at 12,000 rpm. The remaining AA was oxidized by adding excess 30 wt% H_2_O_2_ to the black paste, which was constantly stirred at 60°C, and then centrifuged at 12,000 rpm. Further washing with 70% ethanol followed by distilled water was carried out three times each, and dried for 24 h at 120°C. Using carbodiimide chemistry, the resultant rGO powder after sonication in 0.05 M phosphate buffer (PB, pH 7.5) was labeled with BACE1 Ab. A mixture of equimolar ratio (75 µM) of EDC and NHS was added to 100 μg/ml rGO and incubated for 2 h at room temperature to activate the carboxyl groups on rGO *via* carbodiimide chemistry, which can then react with the amine groups present on the Ab for immobilization forming a covalent bond. This was followed by adding 90 µg of BACE1 Ab dropwise to the activated rGO, incubating at 4°C overnight, and then adding BACE1 Ag. Each synthesis, conjugation, and capture step were thoroughly characterized using Raman spectroscopy, FTIR spectroscopy, EDX, and SEM.

### Fabrication of FTO/rGO/BACE1Ab Electrodes for Analytical Performance and Detection of BACE1 Ag

In this work, 100 μl of activated rGO was allowed to air dry onto the conductive face of an FTO slide at room temperature, followed by carbodiimide immobilization of BACE1 Ab (100 μl). A platinum counter and silver/silver chloride reference electrode were used to carry out the electrochemical experiments for cyclic voltammetry and differential pulse voltammetry. The different stages of fabrication and testing of the modified electrode were confirmed by CV. Different parameters including nanomaterial/Ab concentration, response time, pH, temperature, and rate of scan were standardized for maximum current output *via* CV/DPV. The limit of detection (LOD) was determined form the calibration curve by testing BACE1 Ag samples ranging from 1 fM to 1 µM in 0.05 M PB and spiked serum. 0.1 ml PB/serum samples spiked with various BACE1 Ag concentrations were added to the electrochemical 3-electrode cell containing redox potassium ferri-/ferro-cyanide buffer containing the immersed electrodes. Furthermore, 4-week storage stability as well as 6-cycle repeatability was also evaluated. The specificity was investigated *via* cross-reactivity of the FTO/rGO/BACE1Ab electrode against the NFL protein at 1 μM concentration in the buffer as well as spiked serum and 1X artificial CSF samples. 10X artificial CSF was made by adding 1.25 M NaCl, 260 mM NaHCO_3_, 12.5 mM NaH_2_PO_3_, 25 mM KCl, and 10 mM MgCl_2_ to 1 L double-distilled water. After use, the electrodes were regenerated by rinsing thoroughly with water and storing in a dust-free environment until coating for the next experiment.

## Results and Discussion

### Proof of Principle

**FIGURE 1:**
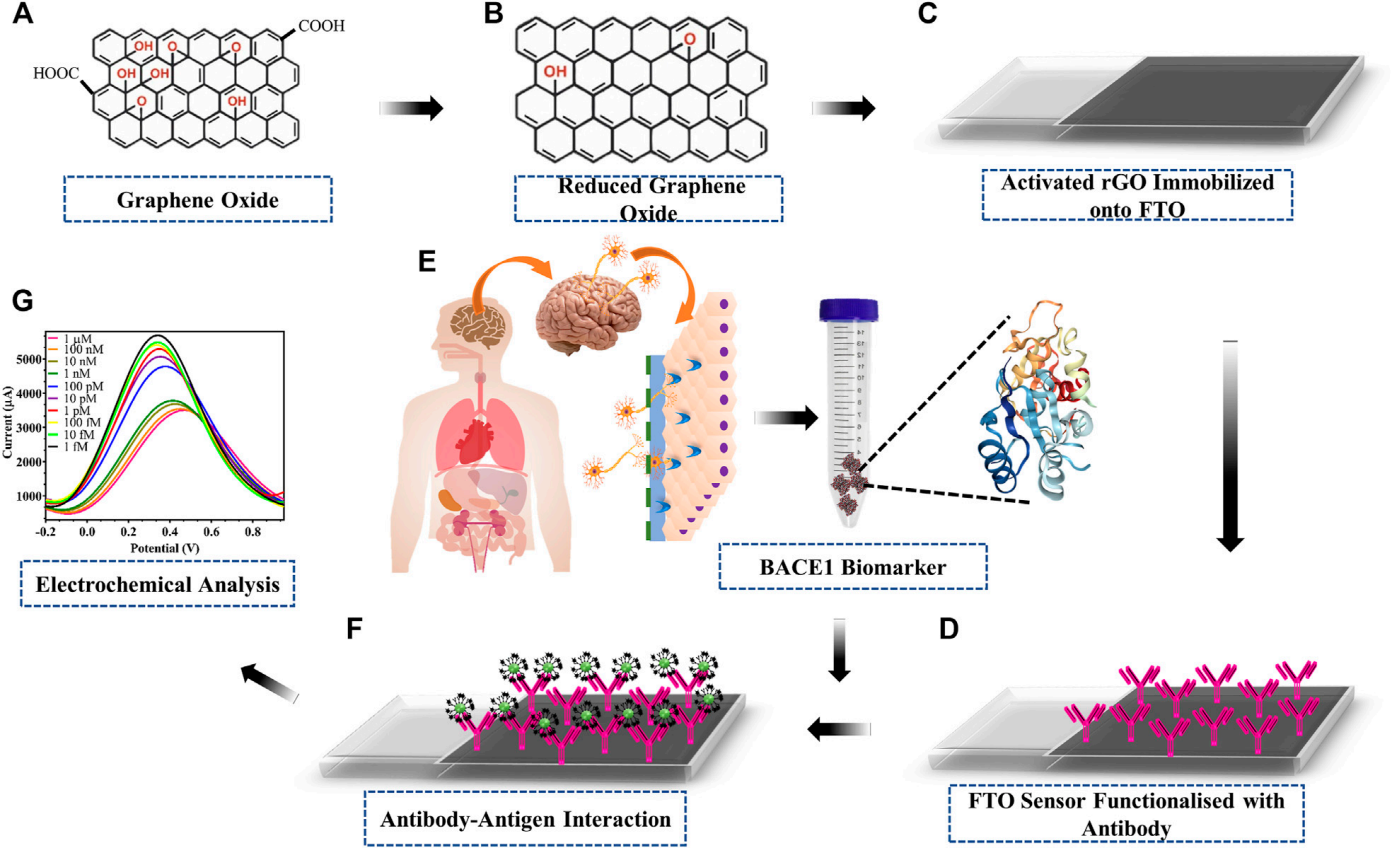
Schematic of fabrication and working of developed electrode: **(A)** GO; **(B)** one-step synthesised rGO; **(C)** activation of rGO via EDC-NHS carbodiimide coupling chemistry coated on the surface of FTO; **(D)** immobilization of BACE1 Ab onto activated rGO; **(E)** Alzheimer’s disease BACE1 biomarker protein sample; **(F)** capture of target BACE1 Ag upon interaction with immobilized BACE1 Ab; **(G)** electrochemical detection of the current output upon Ab–Ag interaction.

The schematic and working principle of the fabricated electrode proposed in this research work is based on electrochemical detection of current variation upon interaction of antibody and antigen, as shown in Figure 1. GO (Figure 1A) was reduced and activated rGO (Figure 1B) was drop cast onto FTO electrodes (Figure 1C). The BACE 1 Ab bioreceptor was immobilized onto the rGO (Figure 1D) using EDC-NHS carbodiimide chemistry, where EDC activated the carboxyl group on rGO by converting the less-reactive carboxyl group into unstable O-acyl-urea derivative, which then reacted with the amine group of NHS to form NHS ester of rGO, which is more stable than the O-acyl-derivative. The NHS ester was then allowed to react with the primary amine group present on BACE1 Ab, resulting in an amide bond between rGO and BACE1 Ab. The fabricated immunosensor was then used to detect BACE 1 Ag (Figure 1E) *via* specific Ag–Ab interaction (Figure 1F) *via* electrochemical analysis (Figure 1G). Hence, the addition of BACE 1 Ag on the FTO/rGO/BACE1Ab-fabricated sensor produced a change in conductivity by inducing variation in the redox potential which was detected by a potentiostat.

### Synthesis, Conjugation, and Characterization of rGO and BACE1 Ab Along with Ag Capture

**FIGURE 2:**
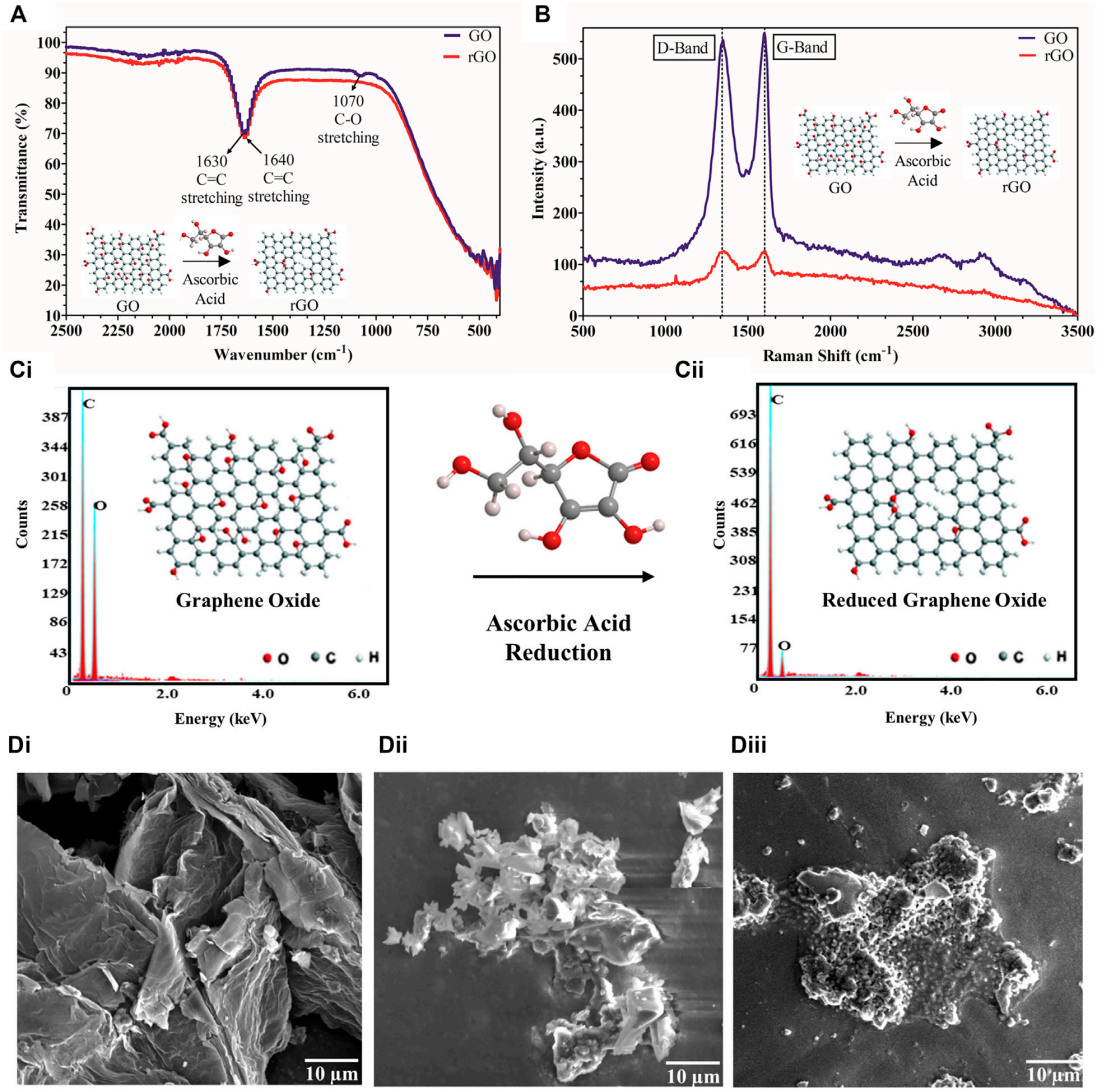
rGO synthesis and conjugation characterization: **(A)** FT-IR spectrum of GO shows peaks at 1,630 cm^−1^ (C = C stretching) and 1,070 cm^−1^ (C-O stretching) whereas rGO shows only a single peak at 160 cm^−1^ (C = C stretching); **(B)** Raman graph showing a higher G-band than D-band by 10 a.u. intensity for GO but no such difference for rGO; **(C)** EDX spectrum showing **(Ci)**. higher amount of oxygen in GO and **(Cii)**. lower amount of oxygen in rGO; **(D)** scanning electron micrographs of **(Di)**. rGO, **(Dii)**. BACE1 Ab deposited on rGO, **(Diii)**. BACE1 Ag bound to BACE1Ab on rGO.

In our work, 1 mg/ml rGO powder, after being synthesized from GO, was suspended to form a uniform black solution upon sonication in 50 mMPB. The FT-IR spectra of GO and rGO are shown in Figure 2A, where defined peaks at 1,630 cm^−1^ (C = C stretching) and 1,070 cm^−1^ (C-O stretching) were observed for GO, whereas only C=C stretching at 1,640 cm^−1^ was seen for rGO due to removal of oxygen groups upon reduction of GO to rGO. rGO synthesis was further observed *via* Raman spectra (Figure 2B), where D-band and G-band appeared at a very similar peak height in case of rGO, but G-band appeared higher than D-band by 10 a.u. intensity in case of GO. The reduction in oxygen upon synthesis of rGO from GO using ascorbic acid was further corroborated by comparing EDX graphs, where reduction in the oxygen content was from 50.7 wt% in the case of GO (Figure 2Ci) to 20.3 wt% in the case of rGO (Figure 2Cii), since rGO contains less oxygen than GO. The morphological studies of the fabrication and working of the electrode were analyzed using SEM. In the corresponding scanning electron micrographs, bare rGO appeared as flakes (Figure 2Di), immobilized BACE1 Ab was observed as white globular structures on the rGO surface (Figure 2Dii), and BACE1 Ag layer captured by the specific Ab was observed (Figure 2Diii).

### Characterization of Optimized FTO/rGO/BACE1Ab Sensor

**FIGURE 3:**
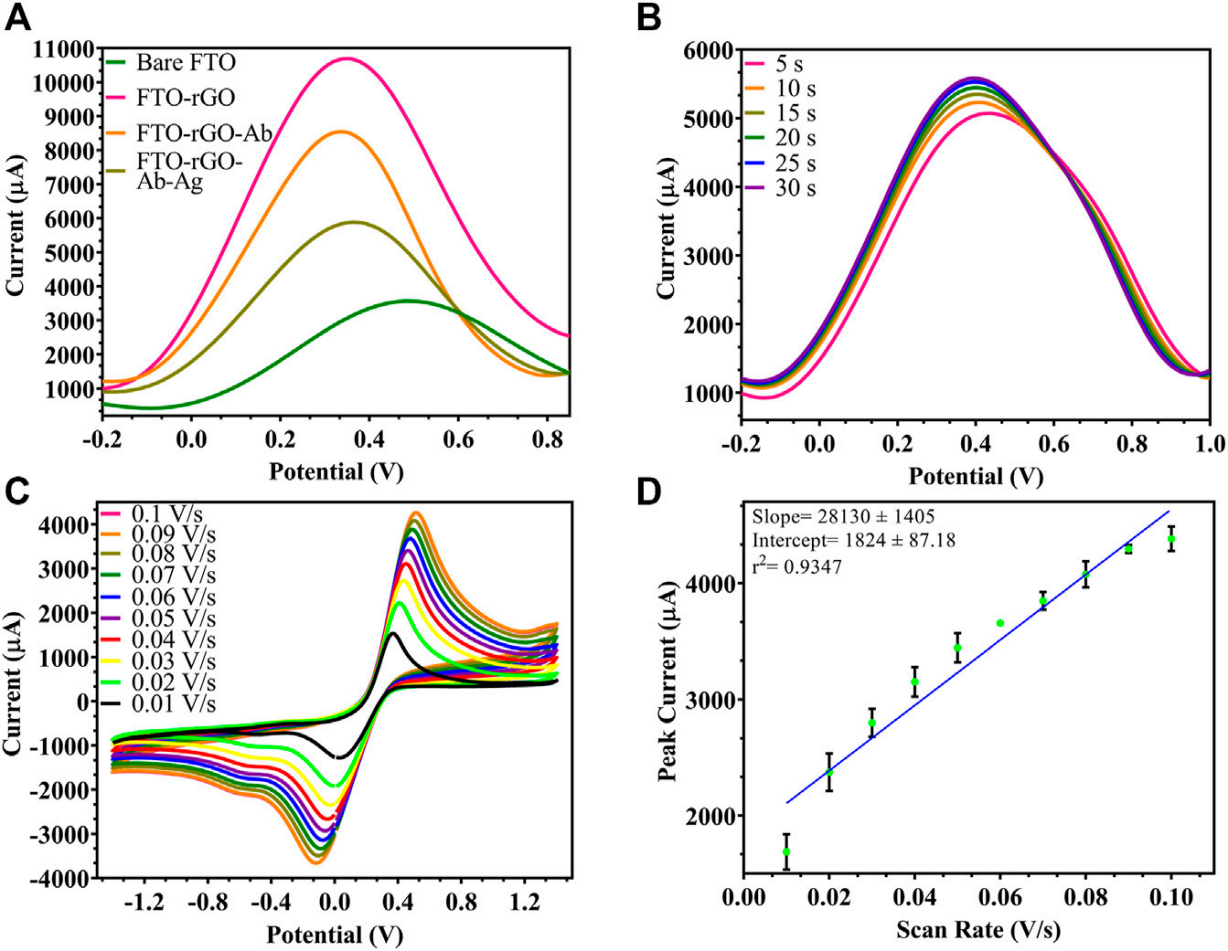
Characterization of the fabricated electrode: **(A)** DPV spectra of fabrication stages, uncoated FTO (3,567 μA), rGO-coated FTO (10696 μA), rGO-BACE1Ab-coated FTO (8,546 μA), Ag captured by rGO-BACE1Ab-coated FTO (5,884 μA); **(B)** DPV of fabricated electrode where response time was tested from 5 s to 30 s, and 5 s and beyond showed stable readings; **(C)** CV of electrode where scan rate was ranged from 0.01 to 0.1 V/s and increase in current was observed; **(D)** linear regression of peak current vs. scan rate.

The electrochemical parameters for the developed electrode were optimized for efficient performance giving maximum current output. CV depends on the redox reaction, while DPV depends on oxidation reaction. As depicted in Figure 3A, DPV of the bare FTO shows a peak current at 3,567 μA, which increased due to oxidation. When the experiment was repeated on a rGO-immobilized FTO electrode, the corresponding peak current increased by 7,129 μA, which corresponds to the conductivity of rGO-enabled increase in surface electron and increased surface area. Following the conjugation of Ab to rGO using EDC-NHS chemistry, a 2,150 μA reduction in peak current was observed, relative to rGO-immobilized electrode. A further 2,662 μA decrease was observed after the attachment of Ag to the Ab. All these are most likely due to the blocking effect of the electron transfer by the proteins^.^ As shown in Figure 3B, among different response times (5-30 s) tested for stable output, 5 s was determined as the least time-point required for a stable response. Since sample preparation/incubation is often required in other BACE1 protein detection techniques, the proposed sensor in comparison gave a stable rapid result consuming less time. The electrode performance was checked within a range of rate of scan from 0.01 to 0.1 V/s (Figure 3C) and reduced current output was seen with reduction in the rate of scan, which was also depicted by a linear regression of peak current vs. scan rate (Figure 3D). As the peak current decreased with scan rate, 0.1 V/s was optimized as the most efficient rate of scan for electrode testing.

**FIGURE 4:**
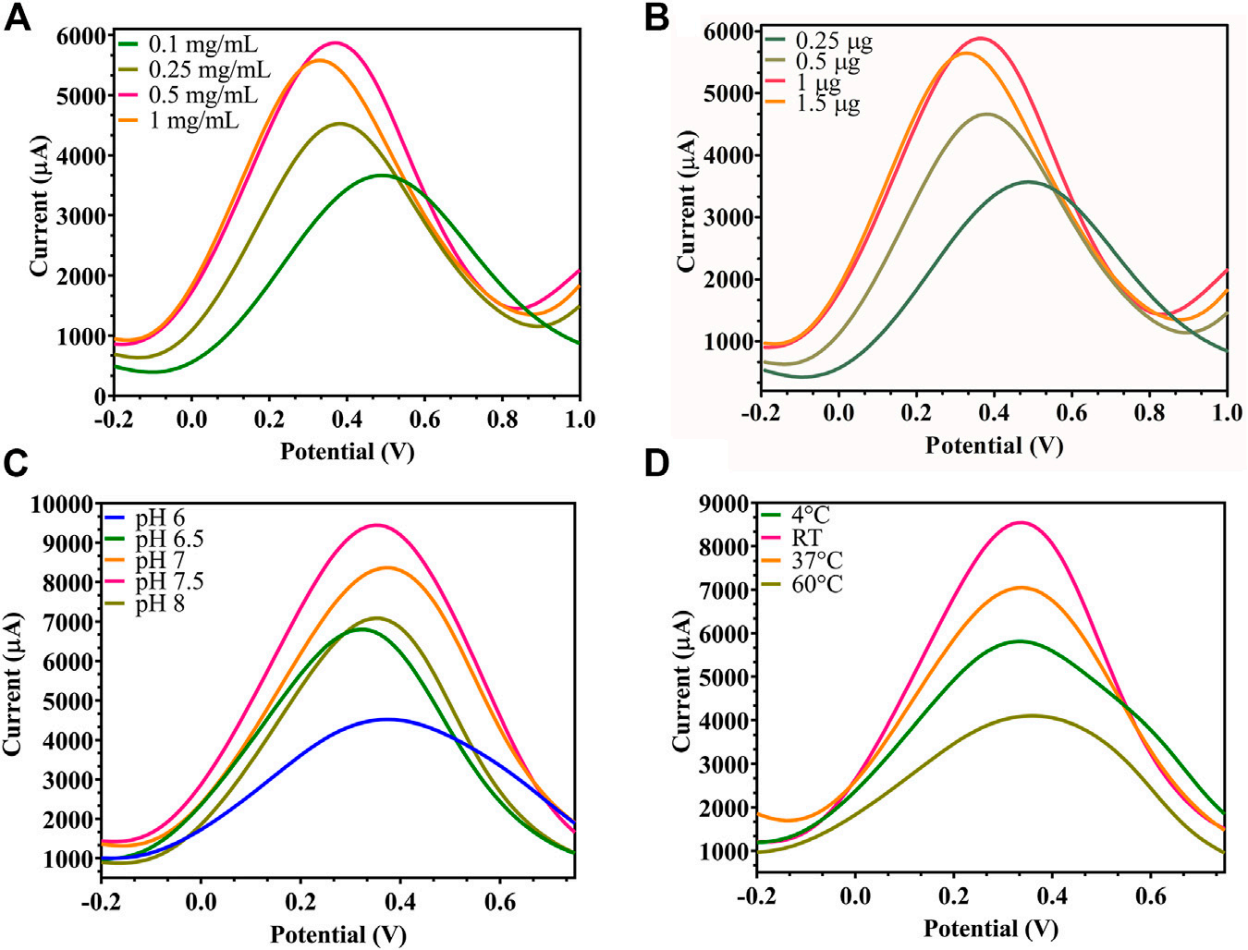
Modified FTO electrode optimization: **(A)** DPV of rGO-immobilized FTO sensor, where the rGO concentration was decreased from 1, 0.5, 0.25 to 0.1 mg/ml, showed maximum signal at 0.5 mg/ml; **(B)** DPV of Ab-immobilized FTO/rGO electrode where BACE1 Ab concentration was decreased from 1.5, 1, 0.5 to 0.25 µg, showed optimum signal at 1 μg; **(C)** DPV of FTO/rGO/BACE1Ab electrode where pH was increased from 6, 6.5, 7, 7.5 to 8, and pH 7.5 gave maximum current output; **(D)** DPV of FTO/rGO/BACE1Ab electrode where temperature was increased from 4°C, RT, 37°C to 45°C, and room temperature showed the highest current output.

By studying the effect of rGO concentration (1, 0.5, 0.24, 0.1 mg/ml) used to prepare the rGO-immobilized electrode, the highest peak current for the oxidation of ferrocyanide was measured at 0.5 mg/ml rGO, as depicted in Figure 4A, since each FTO binding point became saturated at this concentration. Also, the ideal Ab amount to be immobilized on rGO was chosen from varying concentrations (1.5, 1, 0.5, 0.25 µg), and 1 µg resulted in the highest peak current (Figure 4B), since beyond this concentration, all rGO binding sites became saturated with no current increase. While testing environmental parameters, the maximum current output was recorded at pH 7.5 (Figure 4C) and room temperature (RT) (Figure 4D), when the electrode was checked at varying pH (6.0, 6.5, 7.0, 7.5, 8.0) and temperature (4°C, room temperature, 37°C, 45°C), since the immobilized Ab showed maximum activity and did not degrade under these optimum conditions.

### Analytical Output of Modified FTO/rGO/BACE1Ab Sensor for BACE1 Ag Detection

**FIGURE 5:**
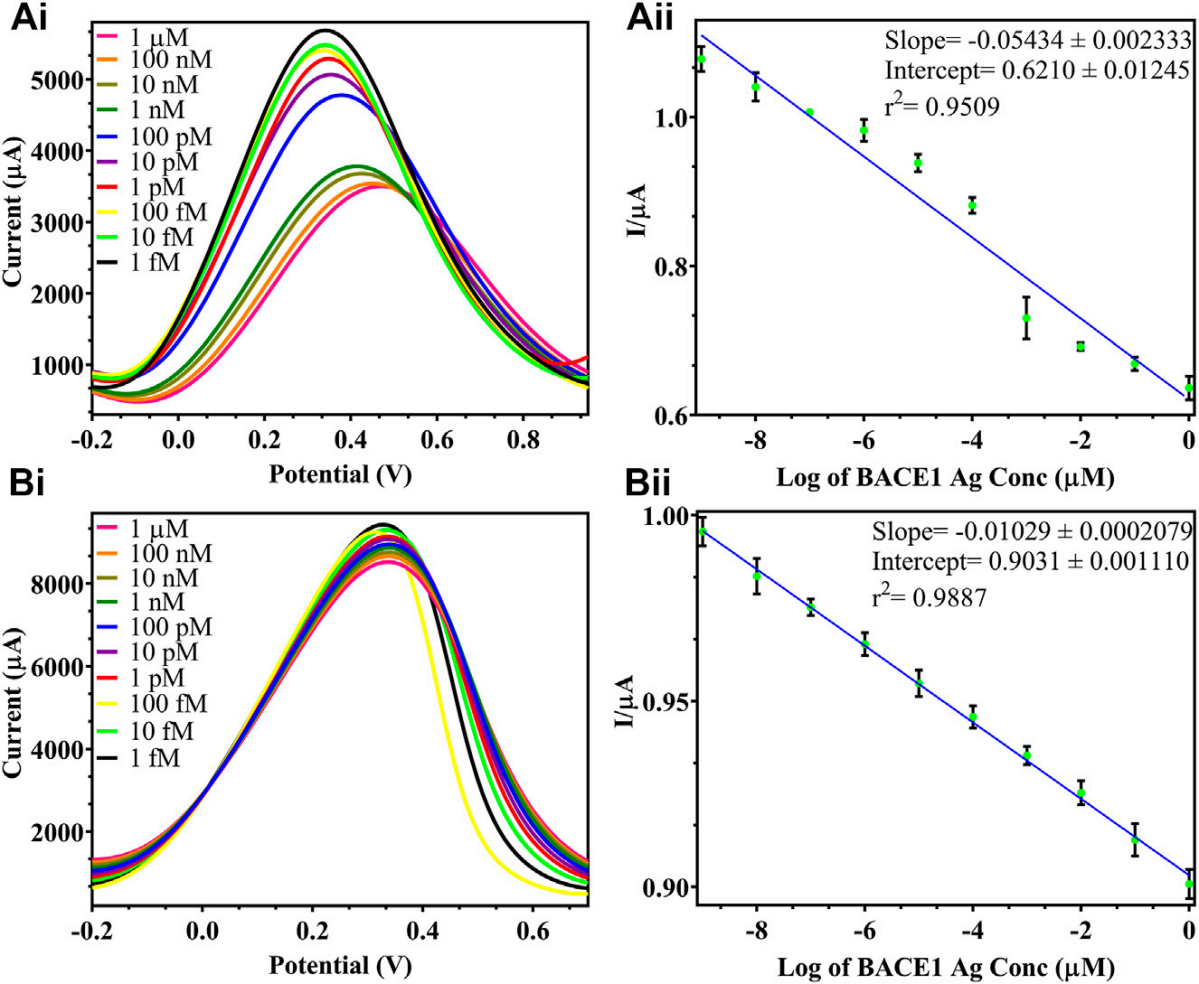
DPV of varying BACE 1 Ag concentrations in buffer samples: **(Ai)**. testing of varying BACE1 Ag concentrations ranging from 1 μM to 1fM at 10-fold dilutions; **(Aii)**. standard calibration graph of log of BACE1 Ag concentrations in buffer vs. I/μA = peak current/blank current; DPV of varying BACE 1 Ag concentrations in spiked serum samples: **(Bi)**. testing of varying BACE1 Ag concentrations ranging from 1 μM to 1fM at 10-fold dilutions; **(Bii)**. standard calibration graph of log of BACE1 Ag concentrations in serum vs. I/μA = peak current/blank current.

DPV was plotted for the detection of BACE1 Ag, and in Figure 5, it was observed that with the increase in concentration of Ag, the current output reduced as added layers of protein result in masking the effect that reduces surface electron transfer. Figure 5Ai shows differential pulse voltammograms of the electrodes tested with increasing concentration of BACE1 Ag in buffer samples from 1 fM to 1 µM. The peak current of the electrodes was observed to decrease as a function of BACE1 Ag concentration. Figure 5Aii shows a plot of the peak current/blank current in Figure 5Ai vs. log of BACE1 Ag concentration in buffer samples, which can be represented by the linear equation shown in Figure 5Aii, where the slope, ordinate intercept, and *r* ^2^ are −0.05434 ± 0.002333, 0.6210 ± 0.01245, and 0.9509, respectively. The *p* value of the calibration graph was significant (*p* value < 0.0001), and hence the regression line was linear. Similarly, Figure 5Bi shows the differential pulse voltammograms of the electrodes tested with increasing concentration of BACE1 Ag spiked in serum samples from 1 fM to 1 µM to verify changes in electrode functioning due to matrix effect. The peak current of the electrodes was again observed to decrease as a function of BACE1 Ag concentration. Figure 5Bii shows a plot of the peak current/blank current in Figure 5Bi vs. log of BACE1 Ag concentration spiked in serum samples, which can be represented by the linear equation shown in Figure 5Bii, where the slope, ordinate intercept, and *r* ^2^ are −0.01029 ± 0.0002079, 0.9031 ± 0.001110, and 0.9887, respectively. The *p* value of the calibration graph was significant (*p* value < 0.0001), and hence the regression line was linear. The LOD in the case of buffer samples was calculated to be 0.64 fM (Figure 5Aii) and 1 fM (Figure 5Bii) in spiked serum. This slight decrease in sensitivity in serum samples is due to the matrix effect caused by other components such as different proteins present in serum, in addition to the target antigen. The formula used to calculate the LOD was 3(S_y_/S), where S_y_ is the standard deviation of response, S is slope, and signal to noise ratio is 3. Since the testing was repeated on multiple electrodes and the average was plotted, the fabricated electrode also showed high reproducibility. Table 1 shows other developed electrochemical immunosensors for the detection of AD, and it can be observed that the LOD of the immunosensor developed in this research work is the lowest, making it the most sensitive electrochemical detection method reported till date.

**TABLE 1:** Comparative data on various electrochemical sensors developed for AD diagnosis for different biomarkers.

S. No.	Type of sensor	Target AD biomarker	LOD	Reference
1	Dual signal hydroxyapatite probe with molybdate (MoO_4_^2-^) NPs and alkaline phosphatase	BACE1 protein	0.1 U/ml	Qu et al. (2016)
3	Graphene field-effect transistor biosensor	Clusterin protein	∼ 300 fg/ml (4 fM)	Bungon et al. (2021)
4	Hydrogel-patterned spiral microelectrode sensor	Amyloid beta 1–40 and 1–42 (Aβ_1-40_ and Aβ_1-42_) peptides	∼ 0.15 pg/ml	Kim et al. (2022)
5	Dual probe on gold nanourchins and nanohorn hybrids	Amyloid-beta (Aβ) peptide	10 fM	Qiu et al. (2021)
6	Electrochemically reduced graphene oxide and gold nanowires on screen-printed carbon electrode	Serum miR-137	1.7 fM	Azimzadeh et al. (2017)
7	Two-photon ratiometric fluorescence resonance energy transfer probe	BACE1 protein	65.3 ± 0.1 pM	Ge et al. (2020)
8	Densely aligned carbon nanotubes multiplexed sensor array	Aβ_42_	2.13 fM	Kim et al. (2020)
		Aβ_40_	2.20 fM	
		t-Tau	2.45 fM	
		p-Tau	2.72 fM	
9	Electrochemical impedance spectroscopy sensor	Tau protein	0.2 μM	Esteves-Villanueva et al. (2014)
10	4 gold microband electrodes self-assembled monolayer and protein G	2N4R tau protein	0.03 pM	Wang et al. (2017)
11	Graphene oxide-polypropylene glycol/anti-tau nano-immunosensor	Tau protein	0.15 nM	Derkus et al. (2017)
12	Gold film- self-assembled monolayers of 3-mercaptopropionic acid sensor	t-Tau protein	NA	Dai et al. (2017)
13	Length-encoded oligonucleotide-aerolysin nanopore-integrated triple-helix molecular switch assay	Tau 381 protein alpha-1 antitrypsin (AAT) protein	6.79 fM	Zou et al. (2019)
		BACE1 protein	77.9 fM	
			86.4 fM	
14	Perylene tetracarboxylic acid/carbon nanotubes ALP–AAT antibody functionalized silver nanoparticles	AAT protein	0.01 pM	Zhu and Lee (2017)
15	Fractal gold nanostructures and enzyme amplification sandwich-type immunosensor	Human apolipoprotein E4 (APOE4)	0.3 ng/ml	Liu et al. (2015)
16	Gold NPs sputtered onto anodic aluminum oxide nano-hemisphere array biochip	Aβ_1–42_	1 pg/ml	Wu et al. (2014)
17	Carbon nanotube film–metal semiconductor field effect transistor	Aβ	1 pg/ml	Oh et al. (2013)
18	Fluorine-doped tin oxide–reduced graphene oxide sensor	BACE1 protein	0.64 fM (buffer) 1 fM (serum)	Current research

### Specificity and Immunosensor Analytical Assay of the Developed FTO/rGO/BACE1Ab Sensor

**FIGURE 6:**
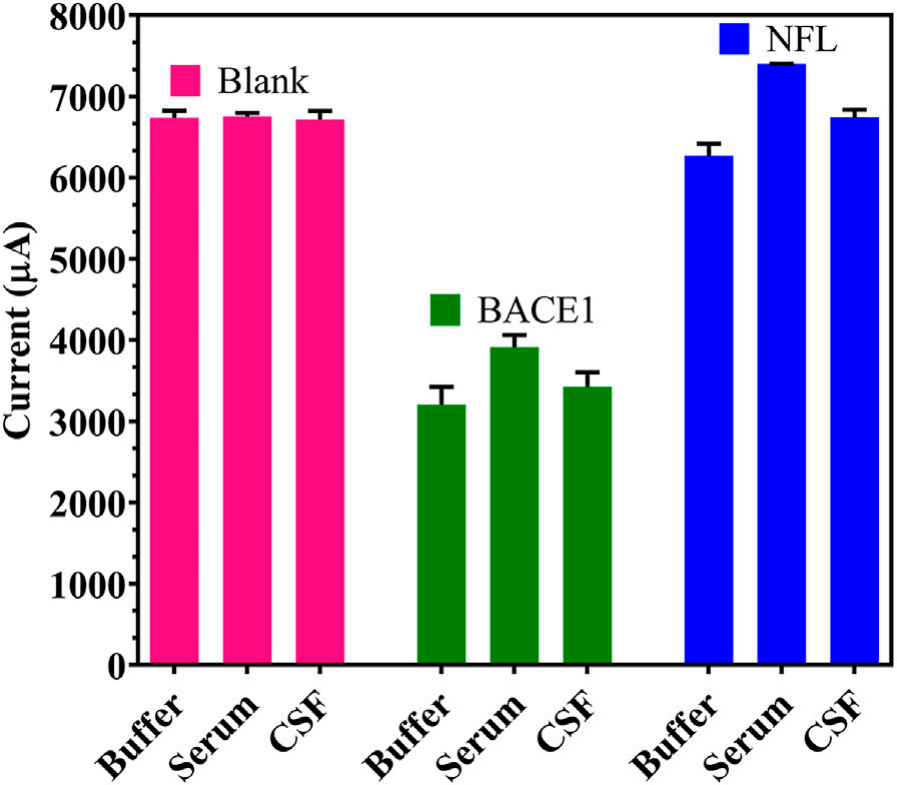
Specificity of the developed immunosensor: Cross-reactivity studies with BACE1 and NFL Ag in spiked buffer, serum and artificial CSF.

Specificity of the fabricated electrode was determined through cross-reactivity experiments to study any non-specific binding of another neuronal Ag. While a reduction in conductivity was observed with BACE1 Ag present in buffer, serum, and artificial CSF samples, no such change was seen on testing with NFL Ag present in buffer, serum, and artificial CSF samples, which showed similar output as the blank samples (non-spiked buffer/serum/artificial CSF), when both Ag was spiked at a maximum concentration of 1 μM (Figure 6).

**FIGURE 7:**
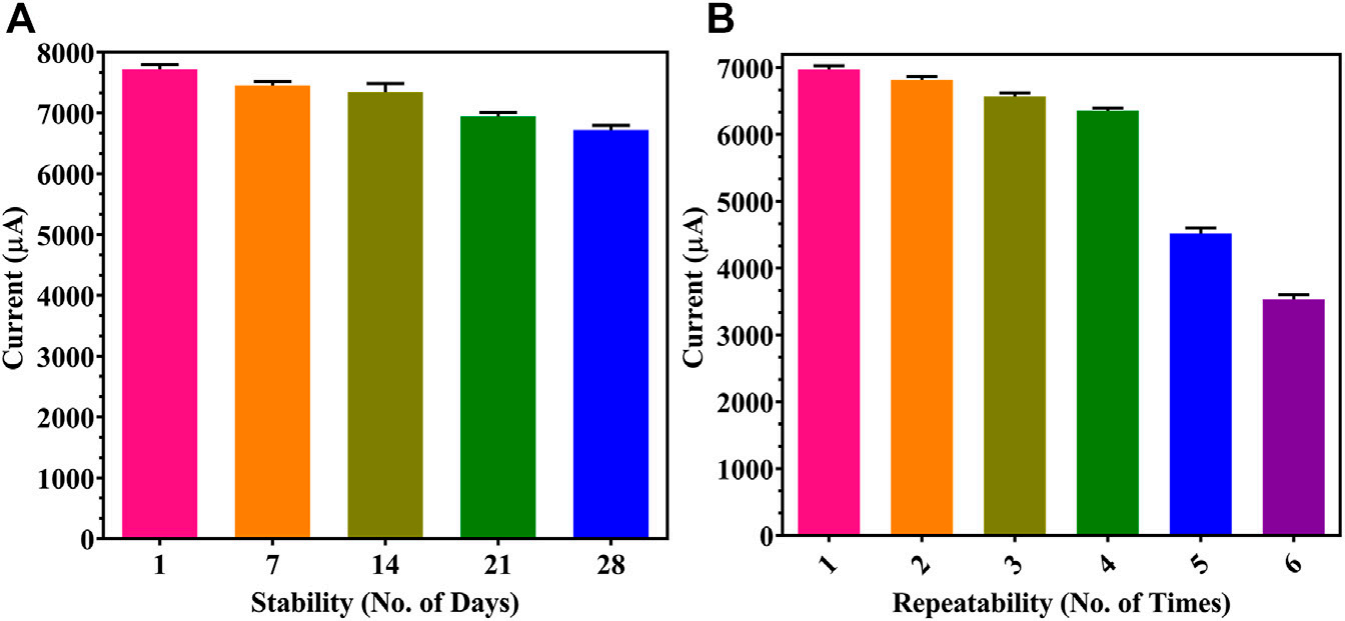
Immunosensor performance: **(A)** storage stability for 1 month at 1-week gaps; **(B)** repeatability studies on individual modified FTO electrodes.

Furthermore, storage stability and repeatability of the developed sensor were determined. In Figure 7A, the proposed sensor showed a stable output upon storage for 1 month when tested at 7-day intervals, with a negligible decrease of 401 μA and 225 μA observed in the 3rd and the 4th week, respectively. This showed that the sensor could be kept in a fridge at 4°C for up to a month without affecting the functioning of the electrode as there is no degradation or reduction in the activity of the immobilized Ab. Multiple readings up to 6 times were taken on each individual electrode (Figure 7B), and a decrease of 1839 μA was seen at the fifth trial; hence, a single electrode may be reused 4 times without any compromise in results since the washing of the electrode after every trial may reduce the concentration and activity of the coated rGO and Ab.

## Conclusion

Herein we have fabricated an FTO/rGO/BACE1Ab sensor for rapid BACE1 Ag detection showing an LOD of 0.64 fM (buffer) and 1 fM (spiked serum), ranging from 1 fM to 1 µM. The electrodes showed high specificity due to negligible cross-reactivity with the neuronal Ag and NFL protein in buffer, as well as spiked serum and artificial CSF. A steady response could be detected in 5 s, and the storage shelf life under refrigeration was found to be a month. Furthermore, a single FTO/rGO/BACE1Ab may be reused 4 times without affecting the sensor output, and rGO was a cheap nanomaterial alternative with an easy one-step reduction synthesis process. The fabrication process is simple and does not require as much time as other existing techniques for diagnosis of AD by detecting BACE1 protein in clinical serum/CSF samples. The proposed electrode shows great future application in the detection of infectious diseases by customizing the sensor by modifying it with other bioreceptors onto different nanomaterials to target any other specific analyte/biomarker.

## Data Availability

The original contributions presented in the study are included in the article/Supplementary Material; further inquiries can be directed to the corresponding authors.
